# Grant Report on d-Serine Augmentation of Neuroplasticity-Based Auditory Learning in Schizophrenia ^†^

**Published:** 2020-08-06

**Authors:** Natalie de la Garrigue, Juliana Glasser, Pejman Sehatpour, Dan V. Iosifescu, Elisa Dias, Marlene Carlson, Constance Shope, Tarek Sobeih, Tse-Hwei Choo, Melanie M. Wall, Lawrence S. Kegeles, James Gangwisch, Megan Mayer, Stephanie Brazis, Heloise M. De Baun, Stephanie Wolfer, Dalton Bermudez, Molly Arnold, Danielle Rette, Amir M. Meftah, Melissa Conant, Jeffrey A. Lieberman, Joshua T. Kantrowitz

**Affiliations:** 1 New York State Psychiatric Institute, New York, NY 10032, USA; 2 Columbia University, College of Physicians and Surgeons, New York, NY 10032, USA; 3 Nathan Kline Institute, Orangeburg, NY 10962, USA; 4 NYU Langone Medical Center, New York, NY 10016, USA

**Keywords:** schizophrenia, cognition, auditory remediation, *N*-methyl-d-aspartate-type glutamate receptor, mismatch negativity, auditory theta

## Abstract

We report on the rationale and design of an ongoing NIMH sponsored R61-R33 project in schizophrenia/schizoaffective disorder. This project studies augmenting the efficacy of auditory neuroplasticity cognitive remediation (AudRem) with d-serine, an *N*-methyl-d-aspartate-type glutamate receptor (NMDAR) glycine-site agonist. We operationalize improved (smaller) thresholds in pitch (frequency) between successive auditory stimuli after AudRem as improved plasticity, and mismatch negativity (MMN) and auditory θ as measures of functional target engagement of both NMDAR agonism and plasticity. Previous studies showed that AudRem alone produces significant, but small cognitive improvements, while d-serine alone improves symptoms and MMN. However, the strongest results for plasticity outcomes (improved pitch thresholds, auditory MMN and θ) were found when combining d-serine and AudRem. AudRem improvements correlated with reading and other auditory cognitive tasks, suggesting plasticity improvements are predictive of functionally relevant outcomes.

While d-serine appears to be efficacious for acute AudRem enhancement, the optimal dose remains an open question, as does the ability of combined d-serine + AudRem to produce sustained improvement. In the ongoing R61, 45 schizophrenia patients will be randomized to receive three placebo-controlled, double-blind d-serine + AudRem sessions across three separate 15 subject dose cohorts (80/100/120 mg/kg). Successful completion of the R61 is defined by ≥moderate effect size changes in target engagement and correlation with function, without safety issues. During the three-year R33, we will assess the sustained effects of d-serine + AudRem. In addition to testing a potentially viable treatment, this project will develop a methodology to assess the efficacy of novel NMDAR modulators, using d-serine as a “gold-standard”.

## SIGNIFICANCE

Schizophrenia is associated with general neurocognitive deficits [1–4], along with related sensory level, early auditory processing (EAP) deficits. For example, schizophrenia patients have elevated thresholds for detecting physical differences in auditory stimuli, such as differences in pitch (frequency) between successive tones (e.g., tone matching thresholds: TMT) [5]. In turn, TMT deficits are associated with deficits in more complex information processing [6], such as reading [7] or auditory emotion recognition (AER) [8–10].

In published studies, EAP deficits have primarily been studied cross-sectionally, but schizophrenia patients show related deficits in neuroplasticity [11], defined as “the brainʼs ability to adapt both structural and functional neural properties in response to internal or external stimuli [12]”. While reduced auditory neuroplasticity can lead to reduced ability to benefit from various forms of cognitive remediation [13,14], including auditory neuroplasticity remediation (AudRem), the effects of intervention strategies aimed at remediating neuroplasticity deficits in schizophrenia have been evaluated to only a limited degree.

**Figure 1.**
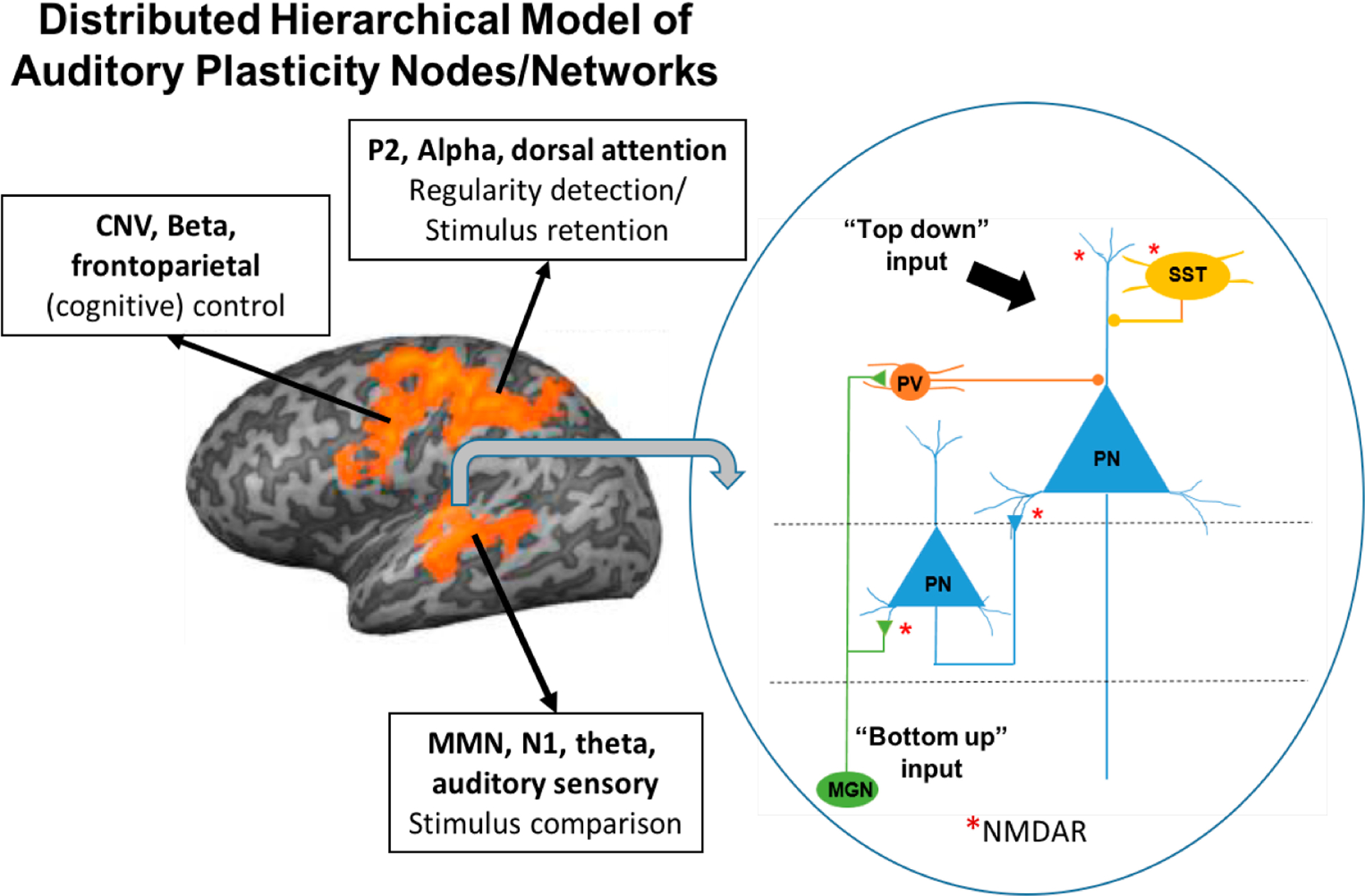
Schematic of model of auditory plasticity in schizophrenia. We show an auditory cortex pyramidal cell receiving bottom-up input from the thalamic medial geniculate nucleus (MGN), parvalbumin (PV), and somatostatin (SST) interneurons, which in-turn receive top-down input from posterior parietal or frontoparietal neurons (inset), thus interacting with dorsal attention and frontoparietal control nodes/networks. NMDAR, noted by the red “*” appear to be involved at multiple levels. Adapted from [15] with permission copyright © 2016 Oxford University Press (For interpretation of the references to color in this figure legend, the reader is referred to the web version of this article).

While the efficacy of cognitive remediation alone is limited [15], as supported by our recently published data [15] and reviews [16,17], our model proposes that localized NMDAR dysfunction leads to impaired auditory neuroplasticity (Figure 1) and that AudRem may be enhanced by concurrent treatment with an *N*-methyl-d-aspartate-type glutamate receptor (NMDAR) modulator. The present project describes an ongoing R61-R33 study focusing on NMDAR based (d-serine) augmentation of auditory learning (pitch discrimination training or AudRem). We operationalize improved (smaller) thresholds for pitch TMT after AudRem as improved plasticity.

In general, brain NMDAR are known to play a crucial role in both neuroplasticity mechanisms [18,19] and the pathophysiology of schizophrenia [20,21]. Moreover, treatment with d-serine, an endogenous agonist for the glycine modulatory site of the NMDAR [22,23], produces intercorrelated improvements in plasticity and the related, neurophysiological EAP measures mismatch negativity (MMN) and auditory θ.

Consistent with the grant mechanism, we are conducting a double blind, placebo controlled, dose-finding study in the R61-phase, assessing the target engagement of d-serine across three separate 80, 100 and 120 mg/kg dose cohorts, followed by an assessment of sustained effects of d-serine in the R33. “Target” refers to a factor that an intervention is intended to modify, leading to improvement in symptoms. Target engagement biomarkers are a measure of the ability of the intervention to “engage” the target. In this study, the target is the NMDAR. Change in threshold for pitch TMT (plasticity), MMN and auditory θ are utilized as both neurophysiological proxies of plasticity and measures of NMDAR target engagement. Detailed rationale is as follows.

### Functional Correlates and Pathophysiology of EAP and Auditory Plasticity in Schizophrenia

Although EAP was once considered an “intact simple function” [24], deficits in pitch processing in schizophrenia were first demonstrated in 1973 in the context of studies of paranoia [5], and have been replicated over recent years by multiple independent groups [25]. EAP deficits are exemplified by elevated TMT thresholds [5,26], and are associated with deficits in more complex information processing that are crucial for economic, occupational, and social functioning for both healthy volunteers [27,28] and schizophrenia [29–31], such as reading [7,32]. EAP deficits also correlate with deficits in global cognition [6,33], AER [8–10,34–37], perceptual music disorders [38,39] and theory of mind [40,41].

As recently reviewed [42], schizophrenia patients show an auditory cortical pattern of EAP deficits. In otherwise healthy controls, isolated bilateral auditory cortex lesions lead to a dramatic increase in pitch TMT. Schizophrenia patients require approximately a 20% difference in pitch (%Δ frequency) to differentiate two tones separated by a brief (300 ms) delay to match performance at 5% Δfrequency in healthy controls [43], a degree of deficit that is as robust (*d* = 1.25) as that typically seen on more complex tasks such as tests of processing speed or executive dysfunction [43]. Consistent with the functional evidence for an auditory cortex deficit in schizophrenia, reduced auditory cortex volume is specifically seen in schizophrenia [44,45]. These structural and functional EAP deficits appear to be NMDAR dependent, as TMT depends on the formation of a NMDAR dependent ʻechoicʼ memory trace.

The auditory cortex is the center of a complex, distributed, hierarchical network [46] (Figure 1), and was traditionally thought to be “fixed” or nonplastic outside of critical periods early in life [47]. Along with other cortical areas [48,49], however, the auditory cortex remains highly plastic into adulthood [50–52]. AudRem is not a simple measure of either sensory learning or TMT, as AudRem can lead to increased recruitment of neurons responding to the training stimuli [53,54], and hence an increase in the so-called cortical “area of representation” of those stimuli and long term auditory neuroplasticity [55]. Thus, our plasticity outcome of improved (smaller) pitch thresholds between successive auditory stimuli after AudRem has direct relevance to cortical neuroplasticity and can facilitate gains in higher-level cognitive processes [56].

### Limitations of Auditory Plasticity Training and Rationale for Combined Treatment

There are no FDA approved pharmacological treatments for cognitive enhancement in schizophrenia, but behavioral interventions have shown promise. Cognitive remediation is commonly defined as “a behavioral training-based intervention that aims to improve cognitive processes [57]”. Cognitive remediation programs vary in the skills they target, with some focused on complex skills like executive functioning, and others targeting more basic skills [58–62]. Posit Science, a program that particularly focuses on AudRem and plasticity, has shown significant, moderate-large (*d* = 0.56–0.86) effect-size improvements in global cognition compared to a videogame control in schizophrenia [63,64]. Subsequent reports using AudRem have been mixed [14,65,66], and furthermore, the clinical burden of a ~50-h treatment administered 3 to 5× a week limits feasibility.

A recently published 12-week study that randomized 103 subjects to either AudRem or a more general cognitive remediation [56] is exemplary of both the promise and limitations of AudRem. At baseline, subjects had clinically significant cognitive deficits, exemplified by a MATRICS [67] composite T score (MCCB) of 20.2. Fifty-three subjects (51.5%) had impaired EAP, defined as a baseline TMT score of <70% correct. EAP deficits were treatable, as within the impaired EAP subgroup, there was a significant, specific effect on auditory cognition (*p* = 0.04, *d* = 0.73). Baseline TMT predicted overall MCCB improvement (*r* = 0.77, *p* < 0.001), suggesting that subjects with baseline EAP deficits will especially benefit from AudRem.

Nonetheless, the efficacy of AudRem on its own is limited. Even in this study [56], overall improvements were small, with subjects improving to a mean MCCB T score of 25. While this exceeds the known practice effects [68] (~2 T score), it remains 2.5 standard deviations below normal (T score = 50). This is consistent with meta-analyses of cognitive remediation trials [57,69] that suggest that up to 45% of people with schizophrenia demonstrate minimal improvement after undergoing a therapeutic dose (≥32 h) of cognitive training [70]. Our group [16,17], and others [12,71–74] have proposed that the efficacy of AudRem may be enhanced by combining with adjunctive medication, particularly an NMDAR modulator. As detailed in the next several sections, NMDAR functioning in schizophrenia is reduced, but not absent. Although the present project is not designed to assess synergistic effects, our model asserts that d-serine combined [71] with AudRem may be an improvement over either one alone.

### Rationale for Studying the NMDAR Target for Enhancing Auditory Neuroplasticity

As previously reviewed [20], in addition to a well-characterized role of brain NMDAR function in the pathophysiology of schizophrenia symptoms [75–77], NMDAR antagonists such as ketamine reproduce core neuropsychological abnormalities of schizophrenia, including auditory cognitive deficits [21,78,79] and MMN [80]. By contrast, similar effects are not observed during exposure to dopaminergic (e.g., methylphenidate [81]) or serotonergic [82,83] agents, further supporting NMDAR models.

The NMDAR is the primary glutamate receptor and facilitates temporal summation of slow excitatory postsynaptic potentials, allowing sustained neural excitation [84]. Thus, NMDAR also have a well characterized role in neuroplasticity, serving as a critical trigger for plasticity-related functions such as acquisition and retention of information [85,86], and long-term potentiation (LTP) and depression (LTD) [84,87,88] throughout the brain. The NMDAR is blocked by a magnesium ion at rest and is dually voltage and ligand gated, and therefore able to detect coincident presynaptic and postsynaptic activity. These unique properties allow the NMDAR to integrate information from multiple pathways and make them critical for memory formation.

As recently reviewed [42], in addition to broader cortical NMDAR deficits, both preclinical and postmortem studies directly implicate NMDAR dysfunction in auditory plasticity deficits in schizophrenia, particularly in impaired pitch processing. Preclinical studies suggest that NMDAR blockade during auditory learning can impair brain plasticity [55,89,90] and NMDAR stimulation produces improvements in both auditory LTP and MMN in healthy rats [91], providing further preclinical support for equating MMN with cortical neuroplasticity.

### Rationale for Early Auditory Processing Electroencephalography (EEG) as a Measure of Target Engagement of NMDAR Agonists

MMN [92,93] is a neurophysiological response elicited most commonly in the context of an auditory oddball paradigm in which a sequence of repetitive standards is interrupted infrequently by a physically different oddball (deviant) stimulus. Deviants may differ from standards in one or more physical and/or abstract dimensions, including pitch or frequency [94,95]. As recently reviewed [42], MMN requires intact auditory cortex functioning, and auditory cortex volume is related to MMN [96]. Support for using MMN as a target engagement biomarker stems from well documented, highly reliable deficits in schizophrenia [33,97–102] and linkages to NMDAR dysfunction in the auditory cortex across rodent [103,104], monkey [105,106] and human [80,107–111] investigations. Moreover, similar to EAP, MMN deficits are highly predictive of poor functional outcomes [9,112–116], as recently confirmed in a large, 1415 subject cross-sectional study of schizophrenia [6]. MMN is increasingly conceptualized as reflecting the “prediction error” evoked when the deviant differs from the standard stimulus [117–121], and thus can also be considered a neurophysiological proxy of plasticity. Recent studies of AudRem in both established [122] and early schizophrenia [115] show that MMN changes are correlated with cognitive improvement.

The utility of MMN as an NMDAR target engagement biomarker is further supported by recent studies showing that MMN deficits in schizophrenia may be sensitive to NMDAR modulating compounds, including d-serine [15,123], glycine [124] and *N*-acetylcysteine [125]. Our recent findings [123,126] suggest that MMN may have positive and negative predictive value in predicting the efficacy of novel NMDAR agonists and plasticity treatment.

In addition to MMN, we will also assess event-related oscillation (ERO) responses as functionally relevant, target engagement biomarkers, particularly auditory θ. Electrophysiological activity is divided conventionally into discrete θ (4–7 Hz), α (7–12 Hz), β (12–24 Hz) and γ (>24 Hz) bands, which reflect differential underlying local-circuit processes [127]. Stimulus-induced responses, including MMN and N1, are typically associated with increases in θ [128]. In contrast to θ and its association with sensory processing, reduction of β activity (termed, event-related desynchronization) has been associated with “higher-level” cognitive processing in frontoparietal networks [129,130]. In our published work, we recently demonstrated that ERO may be especially sensitive to early auditory deficits, and that θ activity during MMN was predictive of symptomatic and functional impairment [128]. Similar findings are seen preclinically [131].

### Specific Rationale for Use of d-Serine to Enhance Plasticity

d-Serine is a direct, full agonist at the d-serine/glycine modulatory site of the NMDAR [22,23]. Activation of the NMDAR requires binding of both glutamate and concurrent binding of d-serine or glycine [132]. Although direct enhancement of NMDAR signaling by glutamate itself can produce excitotoxicity, stimulation via the d-serine site offers a safer method for facilitating activity [20,77]. Furthermore, the d-serine site is not fully saturated in cortical/subcortical regions [133], suggesting that exogenous d-serine may be beneficial. In addition to its more general role in NMDAR modulation, d-serine also has a specific role in LTP/LTD and long-term plasticity [134,135] and synaptogenesis [136]. Schizophrenia patients have well documented, functionally relevant deficits in d-serine [137,138].

Specific advantages of d-serine as a plasticity enhancer include well classified pharmacokinetics (PK) [123,138], and a short half-life (tmax ~30–60 min) allowing for practical administration ~30 min before sessions, thus allowing for plasticity assessment during peak levels. d-serine has shown efficacy in enhancing plasticity outside of critical periods early in life [47], including studies showing efficacy of d-serine in geriatric animals [139] and geriatric [140] and adult healthy volunteers [141]. Chronic use of d-serine does not lead to tachyphylaxis [15,123,142,143], suggesting utility in repeated use, which is essential for a sustained AudRem study. A recent report [72] suggests a specific relationship between d-serine and AudRem, finding a positive correlation between increased d-serine levels and improved global and auditory cognition within the active AudRem group, but not in the sham AudRem group.

The majority of d-serine studies have used a low (30 mg/kg, ~2 g/day) dosage, with a significant, but small effect size improvement at this dose in meta-analyses [137]. This provides proof of concept, but suggests 30 mg/kg may be inadequate to fully engage the target [144,145]. Safety, efficacy and pharmacokinetics of higher dose d-serine (≥60 mg/kg, ≥4 g/day) was recently studied, finding dose-dependent improvement in symptoms and cognition [138]. A significant dose effect for cognition was supported by significantly greater improvement at ≥60 mg/kg vs 30 mg/kg dose for the MCCB composite (*p* = 0.017), with specific improvement in auditory cognition (*p* = 0.035). Pharmacodynamic analysis also supports a dose effect, as higher peak serum levels predicted greater MCCB improvements in this study. Subsequent double-blind studies in both established [123] and early, clinically high risk for schizophrenia groups [146] further support the efficacy of higher dose d-serine. A meta-analysis [123] including high dose studies demonstrates a moderate-large (*d* = 0.7) effect size for negative symptoms, improving on meta-analysis exclusively in low doses.

**Figure 2.**
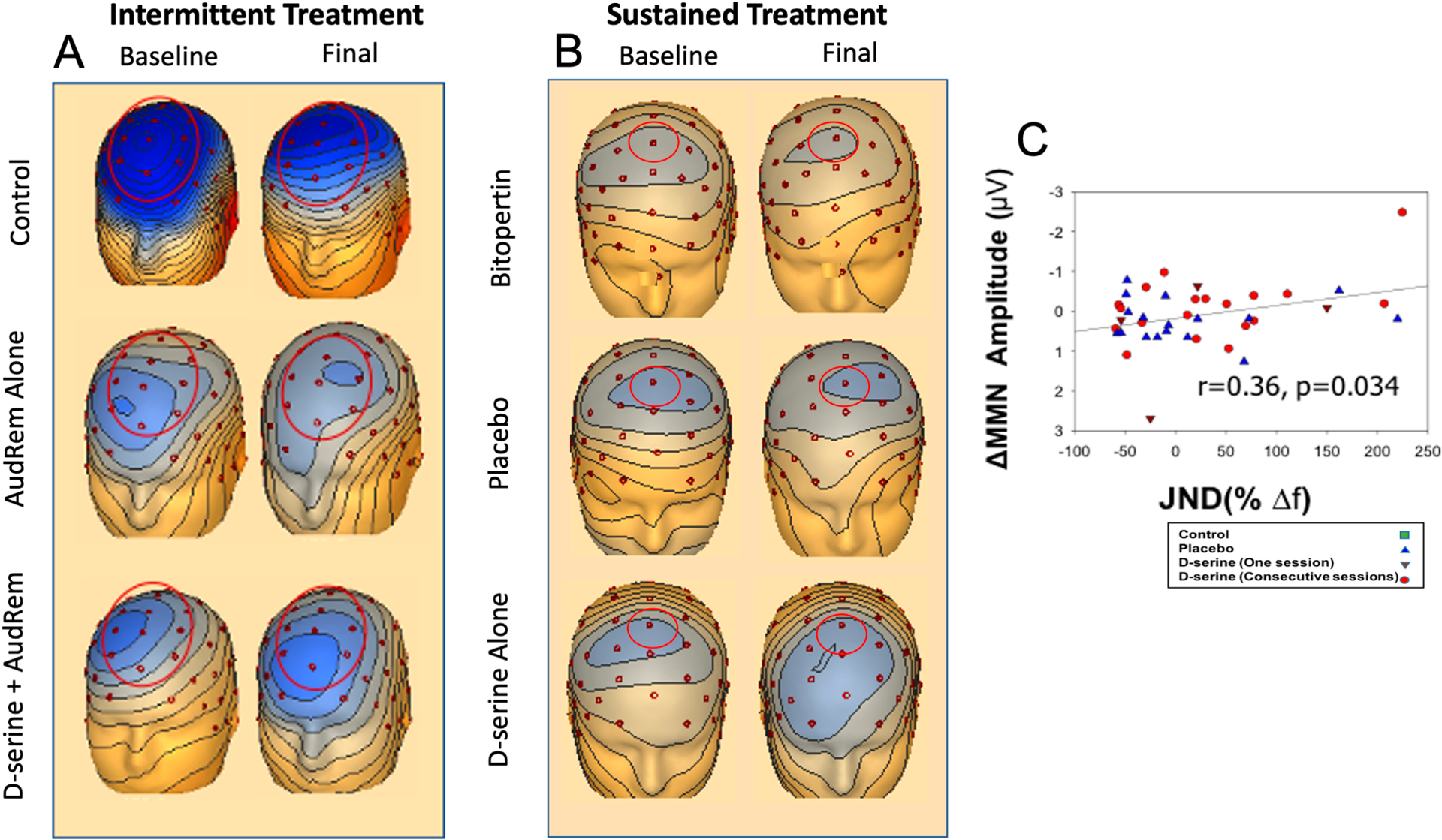
Voltage topography maps for mismatch negativity (MMN) for indicated group for Baseline (left) and Final (right) shown at peak latencies for intermittent (**A**) and sustained (**B**) treatment. Analyzed electrode noted by red circles. Analyzed electrode noted by red circles (Fz). (**C**) Scatter plot for % change in behavioral plasticity during AudRem vs change in MMN amplitude. Modified from [15,124]. (For interpretation of the references to color in this figure legend, the reader is referred to the web version of this article.)

Two recently published studies support the utility of d-serine to study and enhance plasticity and AudRem, finding that both intermittent and sustained d-serine treatment modulates MMN [15,123]. The first [15] serves as the model for the design of the R61–R33 project. In this study, 21 schizophrenia patients received either d-serine 60 mg/kg or placebo with three 1× weekly sessions of AudRem program. ERO responses were recorded during the AudRem, while MMN was measured pre-post training sessions. A significant d-serine treatment effect vs placebo was seen for pitch discrimination after AudRem (behavioral plasticity), along with the EEG plasticity measures MMN (Figure 2A) and activity in the θ and β ranges. Furthermore, following just two d-serine treatments, schizophrenia outcomes for behavioral plasticity were normalized vs. controls. Consistent with prior studies [122], schizophrenia subjects receiving AudRem alone tended to show worsening in MMN. A relationship between improvements in plasticity and MMN was seen (*r* = −0.34, *p* = 0.034, Figure 2C), while changes in θ also correlated significantly with plasticity improvements (*r* = −0.39, *p* = 0.002).

Sustained effects of d-serine on MMN, along with relative effects on NMDAR target engagement has also been recently shown [123,126]. Across two separate, double-blind, placebo controlled, NMDAR-related clinical development programs, MMN showed positive and negative predictive value in predicting efficacy of novel NMDAR agonists. In these studies, 44 individuals with schizophrenia were treated with placebo, d-serine (60 mg/kg/day) or bitopertin, a selective glycine transport type 1 (GlyT1) inhibitor [147] (10 mg) for 4–6 weeks. For d-serine, a significant, large effect size improvement vs. placebo for MMN was seen (Figure 2B), along with intercorrelated improvements in clinical symptoms. By contrast, bitopertin did not significantly affect either symptoms or MMN, consistent with negative Phase II and III studies for bitopertin 10 mg [148,149]. This suggests that bitopertin 10 mg may have failed because of inadequate target engagement, and further emphasizes the need to conduct target engagement studies for dose finding prior to Phase II.

### Limitations of Alternative NMDAR Modulators

As recently reviewed [16,17], most prior studies combining NMDAR agonists with cognitive remediation were conducted with d-cycloserine (DCS), a d-serine derivative which is clinically available because of its use as an anti-tuberculosis agent [150], a function which is unrelated to its NMDAR effects. In low doses, DCS acts as an agonist at the same site as d-serine. Recent studies of low dose DCS in healthy volunteers [151–153], anxiety disorders [154], and schizophrenia [155–157] have shown proof of concept efficacy for plasticity enhancement [155–158]. However, meta-analysis [159,160] shows only small effect size improvements (*d* = 0.25), and low dose DCS is a low potency agonist [161], and higher dose DCS acts as an NMDAR antagonist [162]. Thus, DCS may result in receptor desensitization [163] or psychosis in schizophrenia. Other promising alternatives, such as CTP-692 [164], sarcosine [165] and d-amino acid oxidase inhibitors [166,167] are presently unavailable for general study. The NMDAR partial antagonist memantine has inconsistent effects on MMN [168] and cognition [169] that may be due to dopaminergic [170], not glutamatergic mechanisms [16], and thus would not unambiguously assess the NMDAR target. Thus, d-serine balances efficacy, availability and safety, and is thus the best available agent for assessing NMDAR-based plasticity enhancement and target engagement.

### Innovation

This project is innovative in the following ways:

1. We utilize an innovative neuroplasticity-based AudRem program that is sensitive to acute plasticity changes (R61), and potentially produces sustained improvement (R33).
2. We utilize innovative assessments of NMDAR and plasticity target engagement.
3. We utilize an innovative, weekly dosing strategy to assess the optimal dose (80 vs 100 vs 120 mg/kg) of d-serine.

## APPROACH

This project is a two-phase, two-site study conducted at the Columbia Schizophrenia Research Center at the New York State Psychiatric Institute (Columbia/NYSPI) and the Nathan Kline Institute for Psychiatric Research (NKI).

The first phase (R61), which is ongoing, is designed to assess whether d-serine has dose dependent target engagement over 3 sessions (1× week) of AudRem. In the second phase (R33), we will directly evaluate functional improvement as an outcome, assessing the sustained effects of d-serine plus 16 sessions (1× week) of the same AudRem used in the R61, which has demonstrated a dynamic, direct link between behavioral/neurophysiological plasticity and cognitive improvements. As the structure of grant requires successful completion of R61 phase over 2 years prior to finalization of the R33 design, we do not present the R33 design in detail.

Dr. Joshua Kantrowitz is the overall principal investigator. Dr. Daniel Iosifescu is a study co-investigator and site PI at the NKI site. Overall study coordination is performed by Marlene Carlson, and Constance Shope is the site study coordinator at NKI. Dr. Lawrence Kegeles is a study psychiatrist at Columbia/NYSPI and Dr. Jeffrey Lieberman advises on study implementation. Dr. Pejman Sehatpour directs the neurophysiology laboratory at Columbia/NYSPI and Dr. Elisa Dias directs the neurophysiology laboratory at NKI. Data is managed by the NKI Data Management Center (DMC) (Dr. Tarek Sobeih, director), and uses the Acquire EDC system (http://icrs.rfmh.org). Statistical analysis is performed by Dr. Melanie Wall and Tse-Wei Choo. Study investigators are assisted by a team of research assistants and clinical raters.

The specific aims are as follows:

**Aim #1 (R61):** To determine target engagement and safety of d-serine enhancement of AudRem. 45 schizophrenia patients will be randomized to receive three AudRem sessions plus a double-blind dose of d-serine (80, 100 or 120 mg/kg) or placebo. Based on our data, we hypothesize that d-serine will be safe and lead to greater plasticity, MMN, and θ changes than placebo, with the largest effect at 120 mg/kg.
**Aim #2 (R61):** To confirm the functional relationship of auditory plasticity improvements. In prior studies, auditory plasticity deficits have been related to impairments of higher-level, functionally relevant auditory functions. We hypothesize that plasticity outcomes will be related to functionally relevant outcomes, including auditory cognition and emotion recognition (AER).
**Aim #3 (R33):** To evaluate effects of d-serine-enhanced auditory plasticity on auditory cognition. 60 Schizophrenia patients will be randomized to AudRem plus d-serine or placebo. 16 sessions (1× week) of treatment will be utilized, with dose and final design dependent on R61 results. We hypothesize that d-serine treated subjects will have greater improvements in auditory cognition than placebo. Plasticity, AER, MMN, θ, reading, other cognitive/functional measures, and pharmacodynamics will be secondary outcomes.

### R61 Design

**Figure 3.**
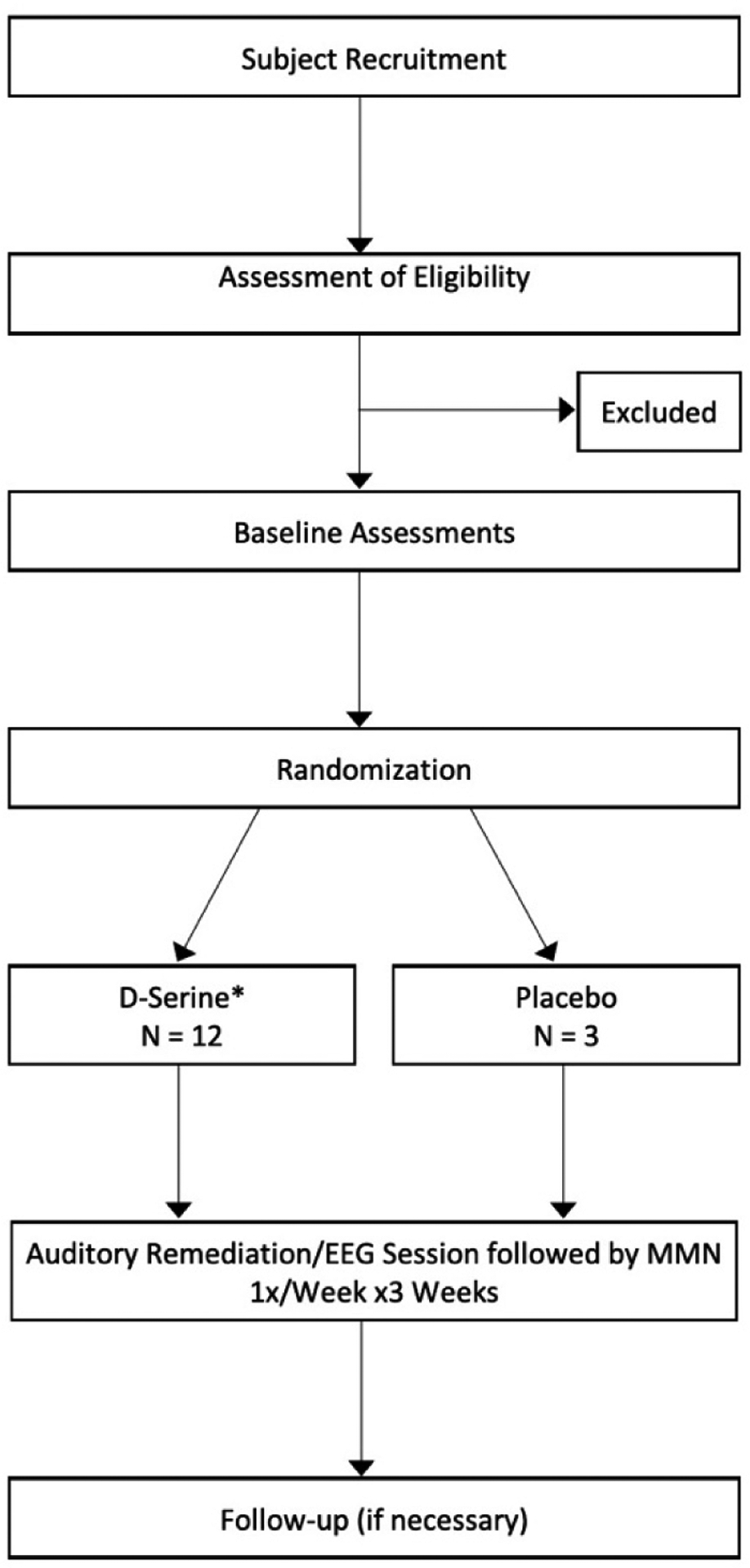
Study Flow Chart. *Three 15-subject cohorts will be conducted sequentially, beginning with a d-Serine dose of 80 mg, followed by a 100 and 120 mg/kg cohort. A 60 mg/kg cohort will be added in case of safety concern in 80 or 100 mg/kg cohort.

The R61 is conducted in cohorts of 15 subjects in which 12 subjects are randomized to double-blind d-serine for each of the three treatment visits and 3 are randomized to double-blind placebo for each of the visits (Figure 3). The first cohort of 15 will receive 80 mg/kg or placebo, the second cohort receive 100 mg/kg or placebo, followed by a third cohort which receive 120 mg/kg or placebo. FDA approval is required after each cohort. Thus far, the first and part of the 2nd cohort have been safely completed.

After informed consent, the subjects undergo full medical and psychiatric screening, which occurs over an up to 31-day period. After satisfying initial inclusion/exclusion criteria (Table 1), subjects complete baseline cognitive and behavioral assessments with a clinical rater.

Subjects are randomized to either receive d-serine or placebo. Subjects will receive the same drug assignment/dose for each of the three treatment days. Randomization is stratified by baseline TMT [26], to allow for potential sub-group analysis. After a negative urine pregnancy test for fecund women and the Columbia Suicide Severity Rating Scale (C-SSRS) [171], each of the three treatment visits begins with a pre-treatment EEG capping. Baseline EEG is collected prior to treatment day 1 only. Subjects then receive d-serine or placebo and AudRem begins 30 min after study drug administration to allow for training during peak d-serine levels. EEG is recorded during sessions to assess ERO, including θ. Immediately after AudRem, subjects complete post-treatment MMN. A d-serine level is drawn after the session using established methods to allow for functional pharmacodynamics readout [138], along with urinalysis and clinical laboratory assessments. The SAFTEE assessment is used to assess general side effects [172].

### Neuroplasticity-Based AudRem Program

We utilize an AudRem program that was originally developed for use in developmental dyslexia [18]. Through 2018, the published studies of this AudRem program include 1137 subjects, including 331 from a patient population (dyslexia: *n* = 237 subjects; schizophrenia: *n* = 61 and ADHD: *n* = 33), amongst 16 publications in total [15,18,173–186]. While most of the replications are in dyslexia by the same group (*n* = 12 published papers), the reliability/validity of the program has been independently replicated by 5 independent groups [15,175,179,184]. Our study [15] was the only one in schizophrenia, and the only one to assess NMDAR mechanisms. While most studies, including our own [15], have focused on acute changes over a few sessions, independently published [180,184] data also show sustained working memory improvements with extended training on similar AudRem programs, supporting our design. In summary, across disorders with impaired auditory plasticity (dyslexia and schizophrenia), all studies that use this or other similar AudRem programs demonstrate the same pattern of improvement over time in behavioral and neurophysiological plasticity.

AudRem sessions are administered once per week ± 2 days. Participants are presented with paired tones (e.g., Stimulus 1 (“reference”) and Stimulus 2 (“test”): S1 and S2) and indicate which tone is higher in pitch (frequency). In the first pair, the between tone ratio is 50% (e.g., 1000 ± 500 Hz), and the difficulty level is adjusted to maintain a steady (~70% correct) level of performance. When the reference (S1) remains constant, highly significant improvement is seen. An added advantage of our AudRem program is that its simplicity minimizes the confound of consolidating a failed or unsuccessful trial, a concern in enhancing extinction learning [187]. EEG analysis will be conducted using previously published methodology [15]. Plasticity will be operationalized as improved (smaller) pitch thresholds between successive auditory stimuli after AudRem.

### Baseline Measures

In order to assess the functional relationships of plasticity, a number of measures will be collected at baseline only, and will be used for correlational analysis under AIM 2 and milestone 2, as detailed below. These measures will be evaluated for use as outcome measures in the R33.

Auditory cognition is defined by the Verbal Memory domain of the MCCB. Cognition is also secondarily assessed using the overall MCCB and remaining individual domains. EAP is assessed with the TMT [35]. The baseline TMT task differs from the primary behavioral plasticity outcome by being a fixed, static measure and does not provide feedback or dynamically change its difficultly based on subject performance. This task consists of pairs of tones, and within each pair, tones are either identical or different in frequency by specified amounts in each of the five blocks (2.5, 5, 10, 20 or 50%).

Reading assessments includes the Wide Range Achievement Test (WRAT) [188], a single-word reading test that assesses premorbid reading level; the Comprehensive Test of Phonological Processing (C-TOPP) [189], which measures phonological processing and the Woodcock Johnson Tests of Achievement, 3rd edition (WJ) [190], which tests comprehension of written language. Social cognition is assessed with the AER [10] task and the Sarcasm task [40]. Psychiatric symptoms are assessed with the Positive and Negative Symptom Scale (PANSS) [191] and general function with the University of California San Diego Performance-Based Skills Assessment (UPSA) [192].

### Study Drug and Maintenance of the Blind

This study is conducted under IND 122821, which specifically allows for the present design and dosing >60 mg/kg. d-Serine or placebo are administered as a solution, prepared (pre-mixed) in water and prescribed only as “study medication”. d-Serine is dosed by weight (e.g., 80 mg/kg for the first cohort) and dispensed to subjects in identical appearing bottles. An artificial sweetener is used as placebo. Medication is dispensed on the day of the visit by unblinded pharmacist who is otherwise uninvolved in the study. Envelopes containing treatment assignment are kept in the pharmacy and are unsealed only in case of medical necessity and blind-breaks will be on an individual subject basis only. The pharmacist does not participate in assessing any dependent variable and conveys no information about drug status to patients or staff except in a medical emergency.

Permitted medications: Subjects will receive concurrent antipsychotics. Patients are allowed to receive the following adjunctive medications during the course of the study: anticholinergic agents; beta-blockers; mood stabilizers, antidepressants; and anti-anxiety agents. As needed doses of clinically determined benzodiazepines or antipsychotics are permitted. Given a possible detrimental effect on cognition, patients are asked to not take these adjunctive medications the night before or on the day of testing/training sessions if clinically feasible.

Plasma d-serine levels: Venous blood samples (10 mL per blood draw) are drawn after the AudRem session for assay.

### Milestones (Go/No Go Criteria)

In the R61-R33 mechanism, emphasis is placed on the development of target engagement biomarkers to ensure that clinical trials produced an adequate test of the underlying hypothesis. The study go/no-go milestones are presented below. Failure to demonstrate target, functional and safety milestones will lead to project termination.

#### Target engagement milestone

The first R61 go/no go criterion is target engagement as demonstrated by a d-serine-induced moderate effect size increase in plasticity, MMN amplitude and θ. Milestones are operationalized as 1: significant (*p* < 0.05) increases in plasticity within d-serine treatment arm(s), and 2: at least a moderate effect size difference (*d* ≥ 0.5) vs placebo treatment for θ, MMN and plasticity. A moderate size difference between active and placebo arms for these three outcomes would indicate that the R33 would have sufficient power to detect a significant effect on the target intervention biomarker, if present.

#### Relationship to function milestone

In addition to demonstration of target engagement, the 2nd R61 Go/No Go criterion is a moderate effect size correlation (*r* = 0.4) between plasticity and baseline auditory cognition. MMN, AER, reading, and other functional measures are secondary outcomes. Establishing this relationship is crucial to demonstrating that changes in AudRem are predictive of sustained functional improvement. A correlation of *r* = 0.4 falls within the range of “medium-large” (*r* = 0.3–0.5) [193].

#### Safety milestone

Nephrotoxicity is a theoretical concern during d-serine treatment, primarily based on studies in rats [194]. d-Serine induced nephrotoxicity, however appears to be isolated to rats, in that other rodent species (e.g., mice, rabbits) to not show similar sensitivity to d-serine, nor is toxicity observed in non-rodent species, e.g., dogs or monkeys [195]. Further, renal side effects of d-serine, even in rats, are fully reversible [194]. The specific sensitivity of rats appears to be due to the presence of a d-serine transporter in rat kidney that actively reabsorbs d-serine from the urine, leading to buildup of high levels within the rat kidney. The presence of this transport mechanism is apparent from the low levels of d-serine in rat urine relative to that of other species, despite relatively similar serum levels [196]. When present in rats, d-serine induced nephrotoxicity leads to a reversible acute tubular necrosis, with high levels of glucose and protein being present in the urine [197]. In humans, d-serine is not actively reabsorbed [196,198], and does not accumulate other than in people with pre-existing renal impairment [199,200]. We are aware of one study that suggested extremely large doses of d-serine can induce nephrotoxicity in a cell culture of human renal tubular cells [201]. However, this study used d-serine concentrations of 10 to 20 mM, which are 10,000 to 20,000 times greater than the expected Cmax in the present study (0.0005 mM or ~500 nM) [138], and thus is of questionable relevance for clinical studies.

15 human trials have been published with d-serine (Table 2), including 451 subjects and treatment duration up to 16 weeks of daily dosing. 122 subjects received high dose (>30 mg/kg), including 16 patients at 120 mg/kg. Across all published studies, only one subject was reported to have abnormal renal values related to d-serine treatment [138]. The abnormality occurred in a subject receiving 4 weeks of the 120 mg/kg dose. Even at that dose, the abnormality was mild in that it involved only an increase in protein (2+ by dipstick) without an accompanying increase in glycosuria, change in creatinine level or other clinical symptoms. The abnormal urinalysis values fully resolved within a few days of stopping treatment. Overall, this 1 case represents 0.2% of all d-serine treated subjects, <1% of subjects treated with continuous high dose d-serine and one of sixteen (6.3%) of subjects treated continuously with 120 mg/kg, emphasizing safety. No renal adverse effects were noted in our prior, 1× weekly intermittent treatment study [15], nor in the ongoing R61. In the present study, overall d-serine exposure is 1/7 of that in prior studies, and thus has a built-in space between doses, maximizing safety.

For the present study, potential nephrotoxicity is monitored through serum chemistry and urine microscopic examination looking for evidence of active sediment (e.g., casts), proteinuria or glycosuria after each dose, as per FDA guidance (Table 3). No subjects with baseline renal impairment, as evidenced by a GFR < 60 or clinically abnormal laboratories during screening labs are enrolled in the study.

### Additional Considerations

Additional measures will be used to inform the design of the R33 study, but not as R61 Go/No-Go criteria. These include (1) relative effects of dose, (2) effect size estimates for secondary EEG outcomes and (3) pharmacodynamics assessment. In general, assuming target engagement, relationship with function and safety milestones are reached by at least one active arm, the dose showing the largest effect size improvement will be utilized for the R33 phase. The 120 mg/kg dose will be used if effect sizes and safety are equivalent between doses.

### Statistical Analysis Plan & Power

Before any specific statistical techniques are applied, we will examine all variables at all time points for illegitimate values, outliers, and other inconsistencies. Distributions of demographic variables and other clinically important baseline variables will be examined and summarized by means, standard deviations, minima, and maxima for continuous measures and proportions for categorical measures. We will make every effort to obtain all data to reduce or eliminate missing data issues. Intent-to-treat analysis will be implemented for all estimation and testing. Tests will be two-sided and statistical significance determined by *p* < 0.05.

### R61 Component

Summaries of clinical and demographic variables will be provided for each of the three cohorts (C1) d-serine 80 mg/kg (*n* = 12) vs placebo (*n* = 3), (C2) d-serine 100mg/kg (*n* = 12) vs placebo (*n* = 3), and (C3) d-serine 120mg/kg (*n* = 12) vs placebo (*n* = 3). As a precaution, any indication of imbalance on important baseline measures between treatment arms (despite randomization) will trigger investigation of whether differences in the primary outcome measures are attributable to these imbalances. For analyses described below, the placebo groups from each of the three cohorts will be combined to yield an effective placebo group of *n* = 9, unless descriptive analyses suggest systematic differences between the three placebo groups, in which case the groups will remain separate in subsequent analyses.

#### Aim 1 Analysis:

Aim 1 hypothesizes that d-serine will lead to greater plasticity, MMN, and θ changes than placebo, with the largest effect being seen at the 120 mg/kg dose. Within-subject changes in plasticity will be examined by using Cohen’s *d* effect size estimates. Similar Cohen’s *d* values will be computed for the combined placebo group. Corresponding 95% confidence intervals (based on standard theory or constructed via bootstrapping if assumptions for constructing the confidence intervals based on parametric methods are not satisfied) will be computed. Similar procedures will be followed for assessing within-subject changes in MMN and θ. For each d-serine dose level, Cohen’s *d* for between-group differences will be computed as (mean change for the d-serine group minus mean change for the placebo group) divided by standard deviation of the change scores in the combined placebo group. Separate analysis will be conducted across and within each d-serine dose group.

#### Aim 2 Analysis:

Aim 2 hypothesizes that plasticity outcomes will be related to (1) functionally relevant outcomes (auditory cognition, primary), (2) emotion recognition (AER), (3) MMN, (4) θ and (5) other functional outcomes and pharmacodynamics. We will examine Pearson correlations (and 95% confidence intervals) between plasticity and measures corresponding to (1)–(5). Spearman correlations (and 95% confidence intervals) will be used in settings where Pearson correlations are determined to be inappropriate.

#### Power Analysis:

The primary results of interest for this R61 are the effect sizes and corresponding 95% confidence intervals, rather than statistical significance testing. Still, we provide some information regarding statistical power for testing within-person change in plasticity (Aim 1) and testing for correlation between plasticity and cognitive function (Aim 2). Using a two-sided paired *t*-test at the 5% significance level, we have >80% power to detect at least *d* = 0.89 for the change in plasticity in any d-serine dose group having *n* = 12 and at least *d* = 1.07 for the change in plasticity in the combined placebo group having *n* = 9. For between group differences, go/no-go milestones are set at an effect size ≥ 0.50. In our published study, an effect size of 0.7 (MMN), 0.79 (θ) and 1.03 (plasticity) was observed using a smaller sample size than is available for the present study. The d-serine dose corresponding to the largest between group effect size with respect to plasticity will be selected as the dose to be used in the R33 trial. In testing for correlation between plasticity and cognition task measures (e.g., auditory cognition) a two-sided t-test for the correlation coefficient using a 5% significance level with 45 subjects (all groups combined) will have >80% power to detect a correlation of ±0.40 or larger in magnitude. We have shown correlations of 0.4–0.62 in our published study [15].

## DISCUSSION

In recent years, numerous promising compounds have failed in Phase III trials. As recently demonstrated [209–213], the working theory behind the R61-R33 mechanism, including the present project, is that prior to the conduct of traditional efficacy studies (Phase II), early-stage (Phase 1b), target engagement biomarker trials should be conducted to assess the both the therapeutic viability of specific compounds and dose range.

We consider the following study outcomes and interpretations as most relevant to further decisions with regard to future development of NMDAR agonist treatment for plasticity deficits. Hypotheses for this project are that (**1**) Treatment with d-serine 120 mg/kg will be safe and lead to greater improvement in plasticity, MMN and θ than lower doses or placebo. (**2**) Plasticity outcomes will be related to functionally relevant outcomes (auditory cognition, primary). (**3**) MMN and θ serve as an effective readouts of NMDAR dysfunction and plasticity in schizophrenia and may serve as effective target engagement biomarkers for putative NMDAR-enhancing treatments.

In addition to MMN and θ, we will also evaluate other time frequency and functional outcomes as secondary measures. The use of MMN and θ is supported by our published studies investigating sensitivity of these measures to d-serine [15,123] and plasticity [122]. By including both target engagement and functional relationship go/no go milestones, successful completion of this project will ensure that d-serine both engages the target and is related to functionally relevant outcomes. In contrast, if both target and functional milestones are not reached, this will fail d-serine as a treatment for plasticity (no go).

The effect size calculation in the present project is based on our published study of d-serine [15], which found large effect sizes from a study of only 21 subjects. Based on these findings, we expect to find at least a moderate (*d* = 0.5) effect size in the proposed R61. In general, effect sizes of “moderate” (*d* = 0.5) are considered “visible to the naked eye of the careful observer”[193] and are widely considered to be the threshold for meaningful clinical effect, and are similar to the significant, moderate-large (*d* = 0.56–0.86) effect-size improvements in global cognition seen with other AudRem programs.

Remediating neuroplasticity is a rate-limiting first step prior to remediating cognition and overall function, and the goal of the present project is to enhance efficacy and efficiency of cognitive, particularly AudRem, fulfilling an unmet clinical need. If successful, this project will develop a “screening” paradigm for assessing the efficacy of a putative cognitive enhancer, stimulating industry involvement with novel NMDAR modulators using d-serine as a “gold-standard.” Positive results will also support a larger, definitive study pairing d-serine itself with other cognitive programs, such as Posit Science, or in alternative dose intervals (1× vs 2× week), a highly innovative and clinically significant outcome. Furthermore, while the present study specifically focuses on auditory plasticity and cognition, a growing literature suggests similar deficits for schizophrenia in the visual system, and thus the present project is relevant across sensory/cognitive domains and across learning disorders and conditions with impaired plasticity (e.g., anxiety disorders or phobia extinction).

## Figures and Tables

**Table 1.** Inclusion and exclusion criteria.

Inclusion Criteria
Age between 18–50
DSM-V diagnosis of schizophrenia or schizoaffective disorder
Auditory cognitive impairment demonstrated by:
MATRICS composite score (MCCB) and verbal memory domain score less than or equal to 0.5 standard deviation below normal (T score ≤ 45) And at least one of the following MCCB verbal memory domain score less than or equal to 0.5 standard deviation below normal (T score ≤ 45) Tone matching score of ≤77.7%
Willing to provide informed consent
Medically stable for study participation
Taking an antipsychotic medication other then clozapine at a stable dose for at least 4 weeks
Judged clinically not to be at significant suicide or violence risk
Clinically stable for 2 months (CGI-S ≤ 4)
Moderate or lower cognitive disorganization (PANSS P2 ≤ 4)
Visual Acuity Corrected to at least 20/30
An estimated Glomerular Filtration Rate (GFR) ≥ 60
Fluent English Speaker
Normal conversational hearing
Willing to use qualified methods of contraception for the study duration and up to 2 months after its end
Exclusion Criteria
Substance abuse (excluding nicotine) within the last 60 days
ECG abnormality that is clinically significant in the context of study participation in the opinion of the study cardiologist
Current clozapine use is excluded for two reasons: to avoid the potential confound of treatment resistant patients and because of clozapine’s intrinsic NMDA agonist properties
Participation in study of investigational medication/device within 4 weeks
Pregnant women or women of childbearing potential, who are either not surgically sterile or for outpatients, using appropriate methods of birth control. Women of childbearing potential must have a negative serum (β-hCG pregnancy test at screening
Presence of positive history of unstable significant medical or neurological illness
Positive toxicology screen for any substances of abuse
Subjects with suicidal ideation with intent or plan (indicated by affirmative answers to items 4 or 5 on the Suicidal Ideation section of the baseline C-SSRS) in the 6 months prior to screening or subjects who represent a significant risk of suicide in the opinion of the investigator

Note: DSM: Diagnostic and Statistical Manual of Mental Disorders; CGI-S: Clinical Global Impression Scale-Severity; PANSS: Positive and Negative Symptoms Scale; C-SSRS: Columbia Suicide Severity Rating Scale; ECG: Electrocardiogram.

**Table 2.** Renal Safety of d-serine.

Reference	Active d-serine *“n”* & diagnosis	Dose	Renal Abnormalities
**High dose**			
[146]	20 CHR (prodrome)	60 mg/kg/day for 16 weeks	None
[15]	21 schizophrenia (Sz)	60 mg/kg single dose once a week	None
[123]	16 Sz	60 mg/kg/day for 6 weeks	None
[202]	10 Sz	3 g/day for 6 weeks (~45 mg/kg)	None
[138]	47 Sz	12 Sz at 30 mg/kg 19 at 60 mg/kg 16 at 120 mg/kg for 4 weeks	1 subject showed 2+ proteinuria without glycosuria after 4 weeks of 120 mg/kg, without change in creatinine. Proteinuria resolved following d-serine discontinuation.
[203]	20 healthy controls	60 mg/kg single dose	None
**Low Dose**			
[204]	14 Sz	30 mg/kg/day for 6 weeks	None
[205]	10 Sz	30 mg/kg/day for 6 weeks	None
[206]	19 Sz	30 mg/kg/day for 6 weeks	None
[207]	21 Sz	2 g/day for 6 weeks (~30 mg/kg)	None
[208]	20 Sz	2 g/day for 6 weeks (~30 mg/kg)	None
[144]	51 Sz	30 mg/kg/day for 12 weeks	None
[145]	97 Sz	2 g/day for 16 weeks (~30 mg/kg)	None
[141]	35 healthy controls	2.1 g single dose (~30 mg/kg)	None
[140]	50 healthy older adults	30 mg/kg single dose	None

**Table 3.** Safety procedures to be performed at every treatment visit, as approved by IND.

Urinalysis with microscopies will be done at every visit. Immediately discontinue d-serine for unexplained serum creatinine increase >0.3 mg/dL over the pre-study value or for >1 granular or muddy casts. Treat as serious adverse event (SAE) possibly related to study medication. Repeat until clear × 2 to demonstrate reversibility Hold d-serine for >1 hyaline casts, and repeat lab. Ask subject to eat more salt and drink more water. If absent on repeat, reinstate D-serine and treat as adverse event (AE). If present on repeat, continue to hold d-serine and repeat lab once again. If still present on second repeat, discontinue d-serine and treat as SAE possibly related to study medication. Repeat until clear × 2 to demonstrate reversibility. Hold d-serine for proteinuria > 100 mg/dL or unexplained glucose >250 g/dL (both equivalent to 2+). If absent on repeat, resume d-serine and treat as AE. If still present on repeat, discontinue d-serine. Repeat until clear × 2 to demonstrate reversibility. This would be treated as SAE possibly related to study medication. Unexplained glycosuria is defined as increased urine glucose in absence of corresponding increase in serum glucose levels, in patients without glycosuria at baseline. Continue d-serine for proteinuria >30 but <100 mg/dL (1+), or unexplained glycosuria (>100 but < 250 g/dL) but repeat. If absent on repeat, continue d-serine and treat as AE. If still present on repeat, hold d-serine and repeat once more. If absent on repeat, resume d-serine and treat as AE. If still present on second repeat, discontinue d-serine and treat as SAE possibly related to study medication. Repeat until clear × 2 to demonstrate reversibility. For other kidney related measures (e.g., ketones, bilirubin, WBC, RBC, bacteria, crystals), repeat, but no need to discontinue even if present on repeat, since unlikely to be d-serine related. Manage in consultation with medical specialist. Contaminated samples (hemolyzed/non-clean catch) will be repeated.


## ABBREVIATIONS

AudRem: Auditory Remediation
NMDAR: *N*-methyl-d-aspartate-type glutamate receptor
MMN: mismatch negativity
EAP: Early Auditory Processing
TMT: Tone Matching Threshold
AER: auditory emotion recognition
ERO: event-related oscillation
MGN: medial geniculate nucleus
PV: parvalbumin
SST: somatostatin interneurons
MCCB: MATRICS
GlyT1: selective glycine transport type 1
DCS: d-cycloserine
NYSPI: New York State Psychiatric Institute
NKI: Nathan Kline Institute for Psychiatric Research
DMC: Data Management Center
C-SSRS: Columbia Suicide Severity Rating Scale
WRAT: Wide Range Achievement Test
C-TOPP: Comprehensive Test of Phonological Processing
WJ: Woodcock Johnson Tests of Achievement, 3rd edition
PANSS: Positive and Negative Symptom Scale (PANSS)
UPSA: University of California San Diego Performance-Based Skills Assessment
